# Electrical Muscle Stimulation: An Effective Form of Exercise and Early Mobilization to Preserve Muscle Strength in Critically Ill Patients

**DOI:** 10.1155/2012/432752

**Published:** 2012-04-01

**Authors:** Eleftherios Karatzanos, Vasiliki Gerovasili, Dimitrios Zervakis, Elli-Sophia Tripodaki, Kleovoulos Apostolou, Ioannis Vasileiadis, Emmanouil Papadopoulos, Georgios Mitsiou, Dimitra Tsimpouki, Christina Routsi, Serafim Nanas

**Affiliations:** First Critical Care Department, Evangelismos Hospital, National and Kapodistrian University of Athens, 106 75 Athens, Greece

## Abstract

*Purpose*. This is a secondary analysis of previously published data to investigate the effects of electrical muscle stimulation (EMS) on strength of various muscle groups in critically ill patients. *Methods*. One hundred forty-two consecutive patients, with APACHE II score ≥ 13, were randomly assigned to the EMS or the control group. EMS sessions were applied daily on vastus lateralis, vastus medialis, and peroneus longus of both lower extremities. Various muscle groups were evaluated with the Medical Research Council (MRC) scale for muscle strength. Handgrip strength assessment was also employed. *Results*. Twenty four patients in the EMS group and 28 patients in the control group were finally evaluated. EMS patients achieved higher MRC scores than controls (*P* ≤ 0.05) in wrist flexion, hip flexion, knee extension, and ankle dorsiflexion. Collectively, the EMS group performed higher (*P* < 0.01) in the legs and overall. Handgrip strength correlated (*P* ≤ 0.01) with the upper and lower extremities' muscle strength and the overall MRC scores. *Conclusions*. EMS has beneficial effects on the strength of critically ill patients mainly affecting muscle groups stimulated, while it may also affect muscle groups not involved presenting itself as a potential effective means of muscle strength preservation and early mobilization in this patient population.

## 1. Introduction

Intensive Care Unit acquired weakness (ICU-AW) is a neuromuscular complication frequently observed in survivors of acute critical illness. It is characterized by profound muscle weakness [1] and is associated with delayed weaning from mechanical ventilation [2]. Risk factors include systemic inflammatory response and sepsis [3, 4], several medications [5], prolonged immobility and bed rest [5], and severity of organ dysfunction [6]. Apart from controlling for potentially reversible risk factors and subsequent adjustment of therapy, no other effective means have been suggested so far for the prevention of ICU-AW.

Prevention of ICU-AW is also related to early mobilization and rehabilitation in the ICU. Recent studies have demonstrated that early mobilization can be safe and feasible, with a potential reduction in short-term physical impairment [7, 8]. However, patient's cooperation is necessary for an essential intervention to be applied.

Electrical muscle stimulation (EMS) is a form of exercise and mobilization that does not require active participation and can be applied to immobilized subjects. EMS has been shown to be beneficial in patients with chronic heart failure (CHF) [9] and chronic obstructive pulmonary disease (COPD) [10, 11], as well as ICU and hospital inpatients [12–14].

We have previously shown that EMS in ICU patients induces a systemic acute effect on the microcirculation of muscles not applied [15] and preserves the mass of muscle groups applied [16]. We have also found that EMS prevents ICU-AW, which was diagnosed in 12.5% of the EMS group in comparison to 39.3% of the control group and results in shorter duration of weaning from mechanical ventilation [17]. However, the EMS effects on the strength of individual muscle groups are not known.

Therefore, the primary aim was the posthoc analysis of recently published data [17] to investigate the effect of EMS on the strength of various muscle groups in critically ill patients. Secondary aims were to establish the relationship between different tests of muscle strength evaluation and to explore the technical issues in relation to muscle strength assessment in the ICU setting.

## 2. Materials and Methods

### 2.1. Patients

All patients consecutively admitted to the multidisciplinary university ICU of Evangelismos Hospital during the study period (September 2007–June 2009) were considered for inclusion. Exclusion criteria were age under 18, pregnancy, obesity (BMI > 35 kg/m^2^), preexisting neuromuscular disease (e.g., myasthenia gravis, Guillain-Barré), diseases with systemic vascular involvement such as lupus erythematosus, technical restrictions that did not allow the implementation of EMS such as bone fractures or skin lesions (e.g., burns), and end-stage malignancy. Patients with cardiac pacemakers and those with an ICU stay of less than 48 hours were also excluded. Patients with the diagnosis of brain death were not considered for inclusion. The study was approved by the Scientific Council and the Ethics Committee of “Evangelismos” Hospital in accordance with the ethical standards set by the Declaration of Helsinki, and written informed consent was given by family members of all the patients included in the study.

### 2.2. Study Design and Randomization

This was a posthoc analysis of a randomized parallel intervention clinical trial already reported [17]. On the second day after admission (24 to 48 hours after admission), patients with acute physiology and chronic health evaluation (APACHE) II score ≥ 13 were randomly assigned to the intervention group (EMS group) or the control group. Randomized stratification was performed upon age (≤ or >50 years of age, which is the median value of our ICU patients' age) and gender (male/female). Patients assigned to the EMS group received daily EMS sessions of both lower extremities starting from the second day after admission until ICU discharge and MRC evaluation. Patients in the control group did not receive sham EMS. EMS was applied in addition to the usual ICU care.

### 2.3. Electrical Muscle Stimulation

EMS (45 Hz, 400 *μ* sec, 12 sec on – 6 sec off, 0.8 sec ramp up/ramp down duration) was implemented simultaneously on vastus lateralis, vastus medialis, and peroneus longus of both lower extremities. After shaving (in the case of hairy skin) and skin cleaning, rectangular electrodes (90 × 50 mm) were placed on the motor points of the aforementioned muscle groups of both legs (Rehab 4 Pro, CEFAR Medical AB, Malmö, Sweden). Amplitude was set at levels able to cause visible contractions. In case of doubt, contraction was confirmed by palpation of the muscles involved. During the session, the angle of the patients' knee joint was approximately 40° (0° corresponds to full knee extension). EMS sessions lasted for 55 min including 5 minutes for warm up and 5 minutes for recovery. The mean number and proportion of sessions that took place was  8 ± 6  and 82 ± 20%.

### 2.4. MRC Muscle Strength Scale

The Medical Research Council (MRC) score for clinical assessment of muscle strength was used for the evaluation of strength and the diagnosis of ICU-AW. MRC scale application has been previously described in detail [17]. In short, after interruption of sedation, MRC was assessed in three muscle groups in all four limbs on the day the patients had a level of consciousness adequate to cooperate. The movements assessed were shoulder abduction, forearm flexion, wrist flexion, hip flexion, knee extension, and ankle dorsiflexion [18]. For the diagnosis of ICU-AW, the cutoff point of 48 was selected [2]. The MRC score evaluation was performed by two independent investigators, not blinded to patients' allocation and familiar with this technique, who provided written MRC scoring for each muscle group. The mean value of the MRC score of the two investigators was used in data analysis. Manual muscle strength testing has been observed to have very good interobserver reliability [19].

### 2.5. Handgrip Application

Handgrip dynamometry (Lafayette 78011, Lafayette Instrument Co. Inc., Lafayette, IN, USA) was applied to a subgroup of consecutive patients to evaluate handgrip strength as an index of upper-limb muscle strength. It was administered immediately after MRC assessment in both hands. Patients were seated nearly upright, positioned at 140° (180° corresponds to the supine position). The patients' arm was positioned at their side, parallel to the sagittal plane of the body, laid against the bed with the elbow at 90°, and supported by the examiner if necessary. These angles were measured and confirmed with a goniometer. During each trial, patients were continuously encouraged to ‘‘squeeze as forcefully as possible” for 4-5 seconds. Five trials were allowed for each hand alternatively, with a pause of 60 sec between each one. The hand with the higher performance was considered for analysis. Once two efforts differed by less than 5% (with a minimum difference of 1 kg), the larger of the two efforts was considered the maximum value and used for the assessment. Before the measurements, patients were familiarized with the procedure. All measurements were performed by the same experienced examiner, who was not blinded to the patients' allocation. Handgrip dynamometry absolute values were also transformed to relative values (% predicted), according to the norms provided by Schlüssel et al. [20].

### 2.6. Statistical Analysis

Power analysis was performed prior to the study initiation, and it was based on a previous epidemiological study from our ICU [4]. Normality of distribution was checked by employing Kolmogorov-Smirnov or Shapiro-Wilk test. Unpaired Student's *t*-test or Mann-Whitney *U* test (in case of not normal distribution) was employed for between-group comparisons. Categorical variables were compared by chi-square test. The Spearman's *r* coefficient was used for correlations. Between-group comparisons in relation to handgrip dynamometry were made with analysis of covariance (ANCOVA) to adjust for age and gender. The ‘‘95% limits of agreement” method was used for comparison of dominant and nondominant handgrip dynamometry. One-way repeated measures analysis of variance (ANOVA) was made to compare handgrip performance over the 5 trials.

MRC measures are reported as median (25th–75th percentiles). All other variables are presented as mean ± SD. *P* values ≤0.05 were considered statistically significant.

## 3. Results

**Figure 1:**
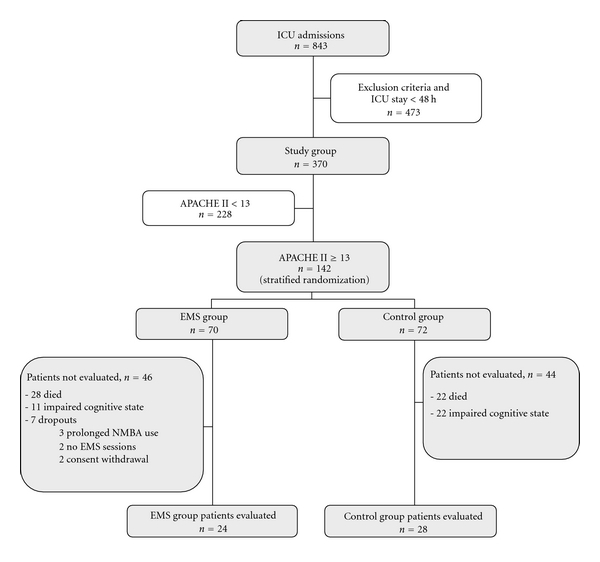
Flow chart diagram of the patients admitted to the ICU during the 30-month study period. NMBAs: neuromuscular blocking agents.

Patient recruitment flow chart, as previously reported [17], is presented in Figure 1. Fifty two patients were finally evaluated, 24 in the EMS group and 28 patients in the control group. Baseline characteristics of all patients in the EMS and control groups have been previously reported [17]. Baseline characteristics of patients finally evaluated in both groups are presented in Table 1.

**Figure 2:**
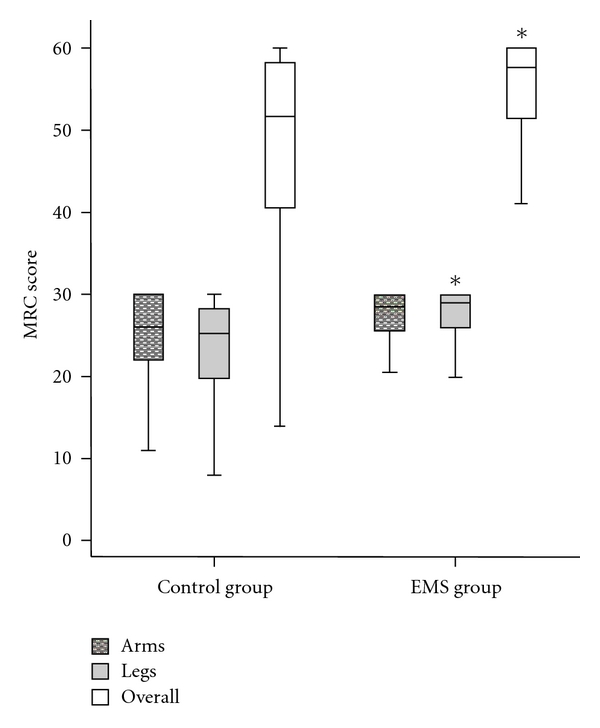
Arms, legs, and overall MRC scores (median, interquartile range) for EMS and control groups. *Significant between-group difference (*P* < 0.05).

 The MRC scores of all the movements of the upper and lower extremities assessed are presented in Table 2. Patients of the EMS group performed statistically higher MRC scores than controls in wrist flexion, hip flexion, knee extension, and ankle dorsiflexion of both sides. No statistical differences were found between the two groups in all other movements. No significant between-group differences were observed for the MRC score of the left and the right arm either, while the MRC scores of the left and right legs were significantly higher in the EMS group. Similar between-group results were found for the total MRC score of the arms (EMS: 28 (26–30); control: 26 (22–30), *P* = 0.16) and the legs (EMS: 29 (26–30); control: 25 (20–28), *P* = 0.01). The overall MRC score was significantly higher in patients assigned to the EMS group in comparison to the control group (58 (51–60) versus 52 (40–58), *P* = 0.04) (Figure 2).

In concern to the baseline characteristics of the subgroup in which handgrip dynamometry was applied (*n* = 21), no difference was found between the control (*n* = 9) and the EMS group (*n* = 12) in gender (males/females, 6/3 versus 10/2, *P* = 0.61), age (65 ± 22 versus 61 ± 14 years, *P* = 0.25), or APACHE II score (19 ± 4 versus 17 ± 4, *P* = 0.46).

No difference was observed between the EMS and the control group in handgrip strength either in absolute (21.4 ± 10.8 versus 14.8 ± 10.7 kg, resp., *P* = 0.18) or relative (60.2 ± 27.3% predicted versus 49.1 ± 28.5% predicted, resp., *P* = 0.38) values. A significant difference was found in handgrip strength between patients diagnosed with ICU-AW in comparison to those without an ICU-AW diagnosis (6.6 ± 4.4 versus 23.4 ± 8.9 kg, *P* < 0.01), even when the comparison was adjusted for gender and age (*P* < 0.01). This was also the case when handgrip performance was expressed in relative values (29.0 ± 22.4% predicted versus 66.0 ± 22.2% predicted, *P* < 0.01).

In concern to between-sides comparison, patients tended to perform better with the dominant rather than the non-dominant hand (16.2 ± 11.1 versus 14.2 ± 9.9 kg, resp., *P* = 0.09). All patients assessed were right handed, therefore the results were similar for comparison between right and left sides. Three patients performed better with the left hand, and in another 3 patients, there was no difference between hands. The 95% limits of agreement were also calculated and found to range from −7.1 to 10.3 kg.

In relation to between-trial comparisons, no difference was observed over the 5 trials performed (1st: 16.1 ± 10.5 kg, 2nd: 16.8 ± 10.8 kg, 3rd: 16.9 ± 11.5 kg, 4th: 16.6 ± 11.0, and 5th: 17.1 ± 11.5 kg, *P* = 0.44).

**Figure 3:**
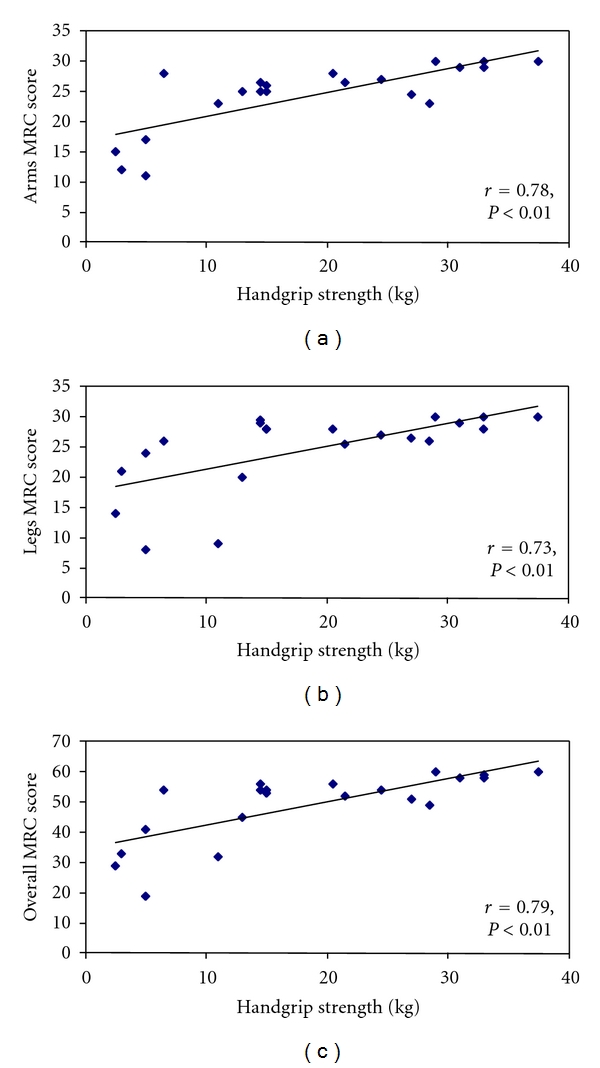
Correlation between handgrip dynamometry performance (in absolute values) and the upper (a), lower (b), and overall (c) MRC scores (*P* < 0.01).

Finally, handgrip strength in absolute values correlated (*P* < 0.01) with the MRC score of upper extremities (*r* = 0.78), lower extremities (*r* = 0.73), and the overall (*r* = 0.79) MRC scores (Figure 3). The respective correlation coefficients (*P* ≤ 0.01) for handgrip strength in relative values (% predicted) were 0.58, 0.53, and 0.55.

## 4. Discussion

The main finding of this posthoc analysis was that EMS resulted in preserved strength, as evaluated with the MRC scale, of directly stimulated muscle groups of the lower extremities. Another finding was that EMS also resulted in preserved strength of the wrist flexors, a muscle group of the upper extremities not stimulated.

### 4.1. EMS and Muscle Strength as Evaluated with the MRC Scale

EMS preserved the strength of quadriceps and the ankle dorsiflexors, which it was applied on. EMS application was directly targeted at vastus medialis and lateralis and longus peroneus, possibly affecting other groups involved in hip flexion and ankle dorsiflexion. The improvement of strength in muscle groups after EMS application has been a consistent finding in CHF and COPD patients [11], COPD [21], and septic patients [22] under mechanical ventilation, as well as healthy populations [23], and could be explained, at least to a large extent, by preservation of muscle mass [12, 16]. ICU patients are characterized by a catabolic state. EMS has been shown to induce an anabolic stimulus in critically ill [14] and postoperative patients [13]. Furthermore, in recently published data of our group, the decrease of the cross-sectional diameter of the rectus femoris and vastus intermedius, a measure of muscle mass evaluated with ultrasonography, was lower in the EMS than the control group after 8-day EMS sessions [16].

EMS also enhanced the strength of wrist flexors, a muscle group involved in upper limb movements, resulting in higher overall MRC score and strength in patients to whom it was applied compared to controls. These findings collectively provide some evidence to imply a systemic effect of EMS in muscle strength of critically ill patients. A likely explanation could be the EMS effects on pathophysiological mechanisms involved in ICU-AW. The fact that EMS affected wrist flexors but not shoulder abductors or forearm flexors suggests some kind of selectivity, which may be related to the size or the characteristics of the muscle groups. The systemic effects of EMS on muscle strength need to be further investigated.

A potential factor relating EMS exercise and effects—local or systemic—in preservation of muscle strength may be inflammation. The latter is associated with the development of ICU-AW [6, 24]. Exercise, in general is known to exert anti-inflammatory effects [25], and this may be the case for EMS [26, 27]. Another factor with a potential role in ICU-AW development, which could be affected by EMS application, is microcirculation [3]. We have previously observed an acute systemic effect of lower extremities EMS on the microcirculation of the thenar muscle, asassessed by near infrared spectroscopy [15]. Karavidas et al. have also observed improved endothelial function in brachial artery after EMS sessions of the legs in CHF patients [27]. Finally, other factors EMS exercise might affect in concern to ICU-AW are mitochondrial function and release of antioxidant enzymes [28, 29], as well as glucose oxidation [30].

EMS, as a possible substitute to aerobic and resistance exercise training in severe CHF and COPD patients, has been shown to improve muscle performance, aerobic exercise capacity, and disease-specific health status [9–11, 21]. EMS application in the ICU setting is directly related to the issue of early rehabilitation and prevention. Recent studies suggest that early rehabilitation is safe and feasible in ICU patients, improving muscle strength, mobilization, aerobic capacity, and ICU and hospital length of stay [7, 8, 31, 32]. However, the vast majority of interventions demand patients' active participation. EMS exercise, in contrast, does not require patients' cooperation and can even be applied to sedated patients.

### 4.2. Muscle Strength as Evaluated with Handgrip Dynamometry

In a subgroup of patients, no differences were observed between the ones treated with EMS and controls in handgrip strength whether expressed in absolute values or % predicted. The sample size however is possibly a confounding factor for definite conclusions, as power analysis suggested.

A significant difference was found in handgrip strength between patients diagnosed with and without ICU-AW, even when results were adjusted for age and gender, factors known to affect strength performance [20]. In a previous study in ICU patients, a similar difference was found between patients with and without ICU-acquired paresis, while handgrip strength was independently associated with hospital mortality [33]. Handgrip strength may be a useful surrogate tool for clinical diagnosis of ICU-AW, as it is easily administered and provides objective and quantified results. Future research is needed for defining proper reference values for ICU-AW diagnosis, according to gender and age.

Standardization of the handgrip measurement is also necessary. In this study, performance of the dominant hand tended to be higher compared to the nondominant hand. In addition, we did not find any differences between the 5 trials. Since the measurement can be affected by movements other than gripping, familiarization was included.

 Handgrip strength correlated to the same extent with the upper limbs, the lower limbs, and the overall MRC score, reflecting the systemic character of ICU-AW and the accompanied muscle weakness. Correlation coefficients of handgrip performance in absolute values are somewhat higher than previously reported [33]; the difference however may be related to the sample size. The fact that correlation coefficients of handgrip expressed in relative values are lower than the respective coefficients in absolute values further emphasizes the necessity for developing norms according to age and gender.

### 4.3. Limitations

 The results of this study are limited by the relatively small number of patients finally able to be evaluated for ICU-AW. This is also a limiting factor in concern to handgrip strength assessment. However, the results are underpowered for definite conclusions. Another limitation is the between-group difference in APACHE II score in admission; however, no differences in other severity scores were observed. Sham-EMS sessions were not applied to the control group, and the MRC scale and handgrip investigators—though independent from each other—were not blinded to patients' group of randomization. Furthermore, ICU staff was not blinded due to absence of sham-EMS sessions, and this may have affected cointerventions. Finally, the results might also have been affected by some between-group differences in diagnosis upon ICU admission.

### 4.4. Clinical Implications

 The results of this study imply that EMS has a beneficial effect in the strength of muscle groups stimulated. EMS of lower extremities may also have a systemic effect in the strength of muscle groups not stimulated and the overall muscle strength of critically ill patients. EMS also prevents ICU-AW development. EMS is safe, well-tolerated and does not require patients' cooperation, thus presenting itself as a potential means for ICU-AW prevention as well as early rehabilitation and mobilization in ICU setting. Handgrip dynamometry, on the other hand, is a simple and easy to administer measurement that could be a surrogate tool in the clinical diagnosis of ICU-AW.

 Future studies should focus on the EMS characteristics able to optimize the effect in critically ill patients. In addition, the effects of EMS on the strength of muscle groups not stimulated, on respiratory muscle strength, and on additional endpoints such as ICU and hospital length of stay, quality of life, and health status after hospital discharge, need to be investigated. Finally, the development of reference values in relation to handgrip dynamometry would potentially aid in ICU-AW diagnosis.

## 5. Conclusions

EMS exercise induces beneficial effects in muscle strength of ICU patients. These effects mainly concern muscle groups directly stimulated, but there is also evidence of effects in muscle groups not stimulated. EMS application constitutes a promising means of muscle strength preservation and early mobilization in critically ill patients (ClinicalTrials.gov number: NCT 00882830).

## Figures and Tables

**Table 1:** Baseline characteristics of the patients finally evaluated in the EMS group and the control group (mean ± SD; in medication variables: median (25th–75th percentiles)).

	EMS group	Control group	*P*
*n*	24	28	
Age, years	55 ± 20	59 ± 21	0.49
Gender, male/female	19/5	22/6	>0.99
SOFA score on admission	8 ± 3	8 ± 3	0.52
APACHE II score on admission	16 ± 4	19 ± 5	0.03
SAPS III score on admission	55 ± 11	58 ± 14	0.34
Diagnostic category at admission			
Brain injury, *n* (%)	9 (38%)	5 (18%)	
Postsurgical, *n* (%)	7 (29%)	5 (18%)	
Respiratory failure, *n* (%)	0 (0%)	2 (6%)	0.05
Sepsis/septic shock, *n* (%)	1 (4%)	10 (36%)	
Trauma, *n* (%)	5 (21%)	5 (18%)	
Other, *n* (%)	2 (8%)	1 (4%)	
Comorbidities			
Cardiovascular disease, *n* (%)	8 (33%)	13 (46%)	0.50
Diabetes mellitus, *n* (%)	3 (13%)	5 (18%)	0.71
GI disease, *n* (%)	3 (13%)	1 (4%)	0.32
Haematologic disease, *n* (%)	1 (4%)	1 (4%)	>0.99
Hepatic disease, *n* (%)	2 (8%)	0 (0%)	0.21
Renal disease, *n* (%)	0 (0%)	6 (21%)	0.03
Respiratory disease, *n* (%)	3 (13%)	9 (32%)	0.18
Other, *n* (%)	2 (8%)	1 (4%)	0.59
None reported, *n* (%)	8 (33%)	8 (29%)	0.95
Sepsis during ICU stay, *n* (%)	16 (67%)	21 (75%)	0.72
Medication			
Sedation, days	5 (2–10)	4 (2–9)	0.85
Aminoglycoside administration, days	0 (0–4)	1 (0–4)	0.18
Corticosteroid administration, days	0 (0–2)	0 (0–2)	0.96
NMBA administration, days	0 (0-0)	0 (0-0)	0.48

EMS: electrical muscle stimulation; SOFA: sequential organ failure assessment; APACHE: acute physiology and chronic health evaluation; SAPS: simplified acute physiology; GI: gastrointestinal; NMBA: neuromuscular blocking agents.

**Table 2:** MRC scores of all the upper and lower extremities' movements (median (25th–75th percentiles)). *P* values refer to between-group comparisons for each movement.

	EMS group	Control group	*P*
Left side			
Shoulder abduction	4 (4-5)	4 (4-5)	0.41
Forearm flexion	5 (4-5)	4 (4-5)	0.26
Wrist flexion	5 (5-5)	5 (4-5)	0.03
Hip flexion	4 (4-5)	4 (3–5)	0.05
Knee extension	5 (5-5)	4 (3–5)	<0.01
Ankle dorsiflexion	5 (5-5)	5 (4-5)	0.04
Upper extremities (in total)	14 (12–15)	13 (11–15)	0.16
Lower extremities (in total)	14 (13–15)	12 (10–15)	0.02

Right side			
Shoulder abduction	4 (4-5)	4 (3–5)	0.36
Forearm flexion	5 (4-5)	4 (4-5)	0.19
Wrist flexion	5 (5-5)	5 (3–5)	0.04
Hip flexion	5 (4-5)	4 (3–5)	0.04
Knee extension	5 (5-5)	4 (3–5)	<0.01
Ankle dorsiflexion	5 (4-5)	5 (4-5)	0.07
Upper extremities (in total)	14 (13–15)	13 (10–15)	0.17
Lower extremities (in total)	15 (13–15)	13 (10–14)	0.02

EMS: electrical muscle stimulation.

## References

[1] Schweickert WD, Hall J (2007). ICU-acquired weakness. *Chest*.

[2] De Jonghe B, Bastuji-Garin S, Sharshar T, Outin H, Brochard L (2004). Does ICU-acquired paresis lengthen weaning from mechanical ventilation?. *Intensive Care Medicine*.

[3] Bolton CF (2005). Neuromuscular manifestations of critical illness. *Muscle and Nerve*.

[4] Nanas S, Kritikos K, Angelopoulos E (2008). Predisposing factors for critical illness polyneuromyopathy in a multidisciplinary intensive care unit. *Acta Neurologica Scandinavica*.

[5] De Jonghe B, Sharshar T, Lefaucheur JP (2002). Paresis acquired in the intensive care unit: a prospective multicenter study. *Journal of the American Medical Association*.

[6] De Letter MACJ, Schmitz PIM, Visser LH (2001). Risk factors for the development of polyneuropathy and myopathy in critically ill patients. *Critical Care Medicine*.

[7] Schweickert WD, Pohlman MC, Pohlman AS (2009). Early physical and occupational therapy in mechanically ventilated, critically ill patients: a randomised controlled trial. *The Lancet*.

[8] Bailey P, Thomsen GE, Spuhler VJ (2007). Early activity is feasible and safe in respiratory failure patients. *Critical Care Medicine*.

[9] Nuhr MJ, Pette D, Berger R (2004). Beneficial effects of chronic low-frequency stimulation of thigh muscles in patients with advanced chronic heart failure. *European Heart Journal*.

[10] Vivodtzev I, Pépin JL, Vottero G (2006). Improvement in quadriceps strenght and dyspnea in daily tasks after 1 month of electrical stimulation in severely deconditioned and malnourished COPD. *Chest*.

[11] Sillen MJH, Speksnijder CM, Eterman RMA (2009). Effects of neuromuscular electrical stimulation of muscles of ambulation in patients with chronic heart failure or COPD: a systematic review of the english-language literature. *Chest*.

[12] Gruther W, Kainberger F, Fialka-Moser V (2010). Effects of neuromuscular electrical stimulation on muscle layer thickness of knee extensor muscles in intensive care unit patients: a pilot study. *Journal of Rehabilitation Medicine*.

[13] Strasser EM, Stättner S, Karner J (2009). Neuromuscular electrical stimulation reduces skeletal muscle protein degradation and stimulates insulin-like growth factors in an age- and current-dependent manner: a randomized, controlled clinical trial in major abdominal surgical patients. *Annals of Surgery*.

[14] Bouletreau P, Patricot MC, Saudin F, Guiraud M, Mathian B (1987). Effects of intermittent electrical stimulations on muscle catabolism in intensive care patients. *Journal of Parenteral and Enteral Nutrition*.

[15] Gerovasili V, Tripodaki E, Karatzanos E (2009). Short-term systemic effect of electrical muscle stimulation in critically ill patients. *Chest*.

[16] Gerovasili V, Stefanidis K, Vitzilaios K (2009). Electrical muscle stimulation preserves the muscle mass of critically ill patients: a randomized study. *Critical Care*.

[17] Routsi C, Gerovasili V, Vasileiadis I (2010). Electrical muscle stimulation prevents critical illness polyneuromyopathy: a randomized parallel intervention trial. *Critical Care*.

[18] Kleyweg RP, Van der Meche FGA, Schmitz PIM (1991). Interobserver agreement in the assessment of muscle strength and functional abilities in Guillain-Barre syndrome. *Muscle and Nerve*.

[19] Fan E, Ciesla ND, Truong AD, Bhoopathi V, Zeger SL, Needham DM (2010). Inter-rater reliability of manual muscle strength testing in ICU survivors and simulated patients. *Intensive Care Medicine*.

[20] Schlüssel MM, dos Anjos LA, de Vasconcellos MTL, Kac G (2008). Reference values of handgrip dynamometry of healthy adults: a population-based study. *Clinical Nutrition*.

[21] Zanotti E, Felicetti G, Maini M, Fracchia C (2003). Peripheral muscle strength training in bed-bound patients with COPD receiving mechanical ventilation: effect of electrical stimulation. *Chest*.

[22] Rodriguez PO, Setten M, Maskin LP Muscle weakness in septic patients requiring mechanical ventilation: protective effect of transcutaneous neuromuscular electrical stimulation.

[23] Bax L, Staes F, Verhagen A (2005). Does neuromuscular electrical stimulation strengthen the quadriceps femoris? A systematic review of randomised controlled trials. *Sports Medicine*.

[24] De Letter MACJ, Van Doorn PA, Savelkoul HFJ (2000). Critical illness polyneuropathy and myopathy (CIPNM): evidence for local immune activation by cytokine-expression in the muscle tissue. *Journal of Neuroimmunology*.

[25] Petersen AMW, Pedersen BK (2005). The anti-inflammatory effect of exercise. *Journal of Applied Physiology*.

[26] Jonsdottir IH, Schjerling P, Ostrowski K, Asp S, Richter EA, Pedersen BK (2000). Muscle contractions induce interleukin-6 mRNA production in rat skeletal muscles. *Journal of Physiology*.

[27] Karavidas AI, Raisakis KG, Parissis JT (2006). Functional electrical stimulation improves endothelial function and reduces peripheral immune responses in patients with chronic heart failure. *European Journal of Cardiovascular Prevention and Rehabilitation*.

[28] da Silva Pimenta A, Lambertucci RH, Gorjão R, dos Reis Silveira L, Curi R (2007). Effect of a single session of electrical stimulation on activity and expression of citrate synthase and antioxidant enzymes in rat soleus muscle. *European Journal of Applied Physiology*.

[29] Gondin J, Brocca L, Bellinzona E (2011). Neuromuscular electrical stimulation training induces atypical adaptations of the human skeletal muscle phenotype: a functional and proteomic analysis. *Journal of Applied Physiology*.

[30] Hamada T, Sasaki H, Hayashi T, Moritani T, Nakao K (2003). Enhancement of whole body glucose uptake during and after human skeletal muscle low-frequency electrical stimulation. *Journal of Applied Physiology*.

[31] Morris PE, Goad A, Thompson C (2008). Early intensive care unit mobility therapy in the treatment of acute respiratory failure. *Critical Care Medicine*.

[32] Burtin C, Clerckx B, Robbeets C (2009). Early exercise in critically ill patients enhances short-term functional recovery. *Critical Care Medicine*.

[33] Ali NA, O’Brien JM, Hoffmann SP (2008). Acquired weakness, handgrip strength, and mortality in critically III patients. *American Journal of Respiratory and Critical Care Medicine*.

